# ﻿Four new species of *Microdochium* (Microdochiaceae, Xylariales) from Hainan, China

**DOI:** 10.3897/mycokeys.126.170451

**Published:** 2025-12-15

**Authors:** Yuxin Shang, Qiyun Liu, Zhaoxue Zhang, Mengyuan Zhang, Zixu Dong, Duhua Li, Yaling Wang, Congcong Ai, Xiuguo Zhang, Jiwen Xia, Zhuang Li

**Affiliations:** 1 Shandong Provincial Key Laboratory for Biology of Vegetable Diseases and Insect Pests, College of Plant Protection, Shandong Agricultural University, Taian, 271018, China Shandong Agricultural University Taian China; 2 College of Life Sciences, Shandong Normal University, Jinan 250358, China Shandong Normal University Jinan China; 3 College of Agriculture and Forestry, Linyi University, Linyi, Shandong, 276000, China Linyi University Linyi China

**Keywords:** *
Microdochium
*, multigene phylogeny, new taxa, taxonomy

## Abstract

*Microdochium* has been reported worldwide as a plant pathogen, endophyte, or saprotroph. This study utilized four genetic loci (ITS, LSU, *rpb2*, and *tub2*) along with morphological characteristics, employing maximum likelihood and Bayesian inference analyses to identify and establish the taxonomic status of four new species from two host plants (Bambusaceae sp. and *Phragmites australis*) in Hainan Province, China. We propose four new species, *Microdochium australiana***sp. nov.**, *M. baishamenense***sp. nov.**, *M. bambusina***sp. nov.**, and *M. bambusarum***sp. nov.** We provide detailed descriptions and micrographs of these species and compare them with other *Microdochium* species.

## ﻿Introduction

*Microdochium* (Microdochiaceae, Xylariales) was established by Sydow with its type species *M. phragmitis*, a fungus from Germany inhabiting living leaves of *Phragmites australis* (Sydow 1924). It belongs to the family Microdochiaceae, proposed by Hernández-Restrepo et al. (2016), which also includes genera such as *Idriella* and *Selenodriella*. Members of this family are characterized by a sexual morph resembling *Monographella*, producing ascocarps and hyaline ascospores, while the asexual morph exhibits multibudded, sympodial, or annellated conidiophores that bear hyaline conidia of various shapes (e.g., cylindrical, fusiform, oval) without accessory structures; some species also form chlamydospores. Although the genus exhibits a cosmopolitan distribution, most records are from Europe and Asia, and approximately 71 species are currently listed in Index Fungorum (http://www.indexfungorum.org/; accessed 30 June 2025). Notably, the morphological characteristics of perithecia, asci, and ascospores show only subtle differences between *Microdochium* and *Monographella* (Amphisphaeriaceae), whereas their anamorphic features are highly similar, leading to frequent historical misclassification of *Microdochium* species within *Monographella* (Parkinson et al. 1981; Samuels and Hallett 1983; Von Arx 1984). Accurate species delimitation within *Microdochium* remains challenging due to insufficient molecular data and morphological convergence among some taxa. Although traditional taxonomy relied heavily on morphological characteristics prior to the adoption of DNA-based phylogenetic analyses (Hibbett et al. 2011), reliable species identification now necessitates the integration of molecular sequence data with detailed morphological characterization.

*Microdochium* species exist not only as plant pathogens but also frequently as endophytes and saprophytes, and they are routinely isolated from a diverse range of plant hosts (Von Arx 1987; Glynn et al. 2005; Jewell and Hsiang 2013; Mandyam et al. 2013; Hiruma et al. 2018; Liang et al. 2019; Lu et al. 2023; Zhang et al. 2023). As pathogenic agents, they primarily affect grasses and cereal crops, causing serious diseases in economically important plants and resulting in yield reductions that lead to substantial economic losses (Gao et al. 2022). In southern China, this genus has been identified as the causal agent of tar spot disease on vetiver grass (*Chrysopogon zizanioides*) (Lu et al. 2023). The disease initially presents as scattered or aggregated black ascomatal stromata on the leaf surface, which are embedded within the leaf tissues (Lu et al. 2023). *Microdochium* species can also colonize plant tissues as endophytes. Many endophytic fungi inhabit plant tissues without causing harm, engaging in mutualistic relationships with their hosts (Rashid et al. 2021). The host provides nutrients, while endophytes enhance plant defense against pests and pathogens and stimulate immune responses (Rashid et al. 2021). Additionally, they suppress pathogenic fungi and promote plant growth (Ali et al. 2024). Certain species within the genus *Microdochium* are also recognized as saprophytes and soil inhabitants (Gao et al. 2022). Saprophytic fungi obtain nutrients from dead organic matter and play a crucial role as decomposers in ecosystems (Saldajeno et al. 2008).

With the advancement of sequencing technologies, phylogenetic studies have transitioned from early single-gene analyses to more comprehensive approaches (Glynn et al. 2005; Jewell and Hsiang 2013; Hiruma et al. 2018; Liang et al. 2019; Liu et al. 2020; Zhang et al. 2023). Hong et al. (2008) employed the ITS sequence to preliminarily examine the phylogenetic relationships among three *Microdochium* species and their type species. Barr (1990) had previously used the LSU sequence to classify the related genus *Monographella* within the Subploasporaceae, a conclusion later revised by Lumbsch and Huhndorf (2010), who reassigned it to the Amphisphaeriaceae.

We identified four novel species of *Microdochium* from specimens collected in Hainan Province, China. Two species were isolated from Bambusaceae and the other two from *Phragmites australis*. Morphological characteristics and molecular sequence data for these species are provided, with taxonomic implications discussed below.

## ﻿Materials and methods

### ﻿Morphological study

Specimens were collected in 2024 from Hainan Province, China, and transported to the laboratory for analysis. Multiple fungal strains were typically obtained from a single specimen, from which pure cultures were subsequently acquired using both single-spore isolation and tissue isolation techniques (Zhang et al. 2025). Tissue fragments (5 × 5 mm) were excised from the edges of leaf lesions and surface-sterilized by sequential immersion in 75% ethanol for 60 s, followed by rinsing in sterile distilled water for 45 s, then immersion in 5% sodium hypochlorite for 45 s, and repeated times in sterile distilled water. The sterilized leaf fragments were blotted dry with sterile paper towels and placed on potato dextrose agar (PDA). All plates were incubated in a biochemical incubator at 25 °C for 3–4 days, after which hyphae were picked from the colony margins and transferred onto new PDA plates. Pure cultures on PDA were incubated at 25 °C for 14 days, with images captured on days 7 and 14 using a Sony Alpha 6400 L digital camera (Sony Group Corporation, Tokyo, Japan). Microscopic examination of fungal structures was performed using an Olympus SZ61 stereomicroscope and an Olympus BX43 compound microscope (Olympus Corporation, Tokyo, Japan), which was equipped with a BioHD-A20c color digital camera (FluoCa Scientific, Shanghai, China) for documentation and image capture of fungal structures. All fungal strains were preserved in sterile 10% glycerol at 4 °C for further studies. Specimens were deposited in the Herbarium of the Department of Plant Pathology, Shandong Agricultural University (HSAUP). Live cultures were stored in the Shandong Agricultural University Culture Collection (SAUCC) and the China General Microbiological Culture Collection Center (CGMCC). Taxonomic information for the new taxa has been submitted to MycoBank (http://www.mycobank.org/, accessed 30 June 2025). The abbreviation of the genus name used in this study is as follows: *M.* = *Microdochium*.

### ﻿DNA extraction, PCR amplification, and sequencing

Genomic DNA was extracted from fungal mycelia scraped from colonies grown on PDA medium using the cetyltrimethylammonium bromide (CTAB) method (Guo et al. 2000; Zhang ang Wang 2020). Four gene regions (ITS, LSU, *rpb2*, and *tub2*) were amplified using the primer pairs listed in Table 1. PCR amplifications were performed in a 25 μL reaction volume using an Eppendorf Master Thermocycler (Hamburg, Germany), containing 12.5 μL of 2 × Hieff Canace® Plus PCR Master Mix (Cat. No. 10154ES03, Yeasen Biotechnology, Shanghai, China), 1 μL each of forward and reverse primers (10 μM, TsingKe, Qingdao, China), 1 μL of template genomic DNA (approximately 10 ng/μL), and double-distilled water to adjust the final volume. PCR products were separated by 1% agarose gel electrophoresis, stained with GelRed, and visualized under ultraviolet light to confirm bands of the expected size. Amplicons were then purified using a gel extraction kit (Cat. AE0101-C; Shandong Sparkjade Biotechnology Co., Ltd., Jinan, China). Bidirectional sequencing was performed by Youkang Company Limited (Zhejiang, China). Consensus sequences were assembled using MEGA v. 7.0 (Kumar et al. 2016). All sequences generated in this study have been deposited in GenBank, with accession numbers provided in Table 2.

**Table 1.** Gene loci and corresponding PCR primers and programs used in this study.

Locus	PCR primers	Sequence (5’ – 3’)	PCR cycles	References
ITS	ITS5	GGA AGT AAA AGT CGT AAC AAG G	(94 °C: 30 s, 55 °C: 30 s, 72 °C: 45 s) × 29 cycles	(White et al. 1990)
	ITS4	TCC TCC GCT TAT TGA TAT GC		
LSU	LR0R	GTA CCC GCT GAA CTT AAG C	(94 °C: 30 s, 48 °C: 50 s, 72 °C: 1 min 30 s) × 35 cycles	(Vilgalys and Hester 1990)
	LR5	TCC TGA GGG AAA CTT CG		
*rpb2*	RPB2-5F2	GGG GWG AYC AGA AGA AGG C	(94 °C: 45 s, 60 °C: 45 s, 72 °C: 2 min) × 5 cycles, (94 °C: 45 s, 54 °C: 45 s, 72 °C: 2 min) × 30 cycles	(Liu et al. 1999; Sung et al. 2007)
	RPB2-7CR	CCC ATR GCT TGY TTR CCC AT		
*tub2*	Btub526-F	CGA GCG YAT GAG YGT YTA CTT	(95 °C: 30 s, 56 °C: 30 s, 72 °C: 45 s) × 35 cycles	(Jewell and Hsiang 2013)
	Btub1332-R	TCA TGT TCT TGG GGT CGA A		

**Table 2.** GenBank accession numbers of the taxa used in the phylogenetic reconstruction.

Species	Culture accession	GenBank accession numbers				Reference
		ITS	LSU	*tub2*	*rpb2*	
*Microdochium albescens*	CBS 243.83	KP858994	KP858930	KP859057	KP859103	(Hernández-Restrepo et al. 2016)
*M. albescens*	CBS 291.79	KP858996	KP858932	KP859059	KP859105	(Hernández-Restrepo et al. 2016)
*M. australiana*	SAUCC 6340-2-6* = CGMCC 3.28622	PQ807110	PV609100	PV686755	PV975978	This study
*M. australiana*	SAUCC 8723-2	PQ807111	PV609101	PV686756	PV975979	This study
*M. bambusae*	SAUCC 1862-1*	OR702567	OR702576	OR715791	OR715785	(Zhang et al. 2023)
*M. bambusae*	SAUCC 1866-1	OR702568	OR702577	OR715792	OR715786	(Zhang et al. 2023)
*M. bambusina*	SAUCC 7531-3* = CGMCC 3.28623	PQ807108	PV609098	PV686753	PV975976	This study
*M. bambusina*	SAUCC 7638-2	PQ807109	PV609099	PV686754	PV975977	This study
*M. bambusarum*	SAUCC 7611-3* = CGMCC 3.28624	PQ807112	PV609102	PV686757	PV975980	This study
*M. bambusarum*	SAUCC 6699-4	PQ807113	PV609103	PV686758	PV975981	This study
*M. baishamenense*	SAUCC 8129-1* = CGMCC 3.28625	PQ807114	PV609104	PV686759	PV975982	This study
*M. baishamenense*	SAUCC 7263-1	PQ807115	PV609105	PV686760	PV975983	This study
*M. bolleyi*	CBS 540.92	KP859010	KP858946	KP859073	KP859119	(Hernández-Restrepo et al. 2016)
*M. chrysanthemoides*	LC5363*	KU746690	KU746736	KU746781	KY883244	(Zhang et al. 2017)
*M. chrysanthemoides*	LC5466	KU746689	KU746735	KU746782	KY883245	(Zhang et al. 2017)
*M. chrysopogonis*	GDMCC 3.683	MT988022	MT988024	MW002441	MW002444	(Lu et al. 2023)
*M. chuxiongense*	YFCC 8794	OK5861616	OK586160	OK556901	OK584019	(Tang et al. 2022)
*M. citrinidiscum*	CBS 109067*	KP859003	KP858939	KP859066	KP859112	(Hernández-Restrepo et al. 2016)
*M. colombiense*	CBS 624.94*	KP858999	KP858935	KP859062	KP859108	(Hernández-Restrepo et al. 2016)
*M. dawsoniorum*	BRIP 65649*	MK966337	ON394569	-	-	(Crous et al. 2020)
*M. dawsoniorum*	BRIP 67439a	MN492650	OM333563	-	ON624208	(Crous et al. 2020)
*M. fisheri*	CBS 242.90	KP859015	KP858951	KP859079	KP859124	(Hernández-Restrepo et al. 2016)
*M. graminearum*	CGMCC 3.23524	OP103965	OP104015	OP242835	OP236026	(Gao et al. 2022)
*M. graminearum*	CGMCC 3.23525*	OP103966	OP104016	OP236029	OP236027	(Gao et al. 2022)
*M. graminis*	ZJE01778*	PP111928	PP111935	PP112588	PP112593	(Yan and Zhang 2024)
*M. graminis*	B13	HQ696038	-	-	-	(Shen et al. 2014)
*M. graminis*	PE110	JX875927	-	-	-	(Shen et al. 2012)
*M. gongcheniae*	YNE01155	PP111925	PP111932	PP112585	-	(Yan and Zhang 2024)
*M. gongcheniae*	YNE01164*	PP111926	PP111933	PP112586	-	(Yan and Zhang 2024)
*M. hainanense*	SAUCC 210781*	OM956295	OM959323	OM981146	OM981153	(Liu et al. 2022)
*M. hainanense*	SAUCC 210782	OM956296	OM959324	OM981147	OM981154	(Liu et al. 2022)
*M. hongkuii*	YNE00384	PP111922	PP111929	PP112582	PP112589	(Yan and Zhang 2024)
*M. hongkuii*	YNE00483*	PP111923	PP111930	PP112583	-	(Yan and Zhang 2024)
*M. hongkuii*	N115	MK304137	-	-	-	From NCBI
*M. indocalami*	SAUCC 1016*	MT199884	MT199878	MT435653	MT510550	(Huang et al. 2020)
*M. insulare*	BRIP 75114a	OQ917075	OQ892168	-	OQ889560	(Tan and Shivas 2023a)
*M. lycopodinum*	CBS 109397	KP859004	KP858940	KP859067	KP859113	(Hernández-Restrepo et al. 2016)
*M. lycopodinum*	CBS 109398	KP859005	KP858941	KP859068	KP859114	(Hernández-Restrepo et al. 2016)
*M. lycopodinum*	CBS 125585*	KP859016	KP858952	KP859080	KP859125	(Hernández-Restrepo et al. 2016)
*M. maculosum*	COAD 3358*	OK966954	OK966953	-	OL310501	(Crous et al. 2021a, b)
*M. majus*	CBS 741.79	KP859001	KP858937	KP859064	KP859110	(Hernández-Restrepo et al. 2016)
*M. miscanthi*	SAUCC 211092*	OM956214	OM957532	OM981141	OM981148	(Liu et al. 2022)
*M. miscanthi*	SAUCC 211093	OM956215	OM957533	OM981142	OM981149	(Liu et al. 2022)
*M. musae*	CBS 143499	MH107894	MH107941	MH108040	-	(Crous et al. 2018)
*M. musae*	CBS 143500*	MH107895	MH107942	-	MH108003	(Crous et al. 2018)
*M. nannuoshanense*	SAUCC 2450-1*	OR702569	OR702578	OR715793	OR715787	(Zhang et al. 2023)
*M. nannuoshanense*	SAUCC 2450-3	OR702570	OR702579	OR715794	OR715788	(Zhang et al. 2023)
*M. neoqueenslandicum*	CBS 108926*	KP859002	KP858938	KP859065	KP859111	(Hernández-Restrepo et al. 2016)
*M. neoqueenslandicum*	CBS 445.95	KP858997	KP858933	KP859060	KP859106	(Hernández-Restrepo et al. 2016)
*M. nivale*	CBS 116205*	KP859008	KP858944	KP859071	KP859117	(Hernández-Restrepo et al. 2016)
*M. nivale*	CBS 288.50	MH856626	MH868135	-	-	(Vu et al. 2019)
*M. novae-zelandiae*	CBS 143847*	LT990655	LT990627	LT990608	LT990641	(Marin-Felix et al. 2019)
*M. novae-zelandiae*	CPC 29693	LT990656	LT990628	LT990609	LT990642	(Marin-Felix et al. 2019)
*M. paspali*	CBS 138620*	KJ569513	-	KJ569518	-	(Zhang et al. 2015)
*M. paspali*	QH-BA-48	KJ569510	-	KJ569515	-	(Zhang et al. 2015)
*M. phyllosaprophyticum*	SAUCC 3583-1*	OR702571	OR702580	OR715795	OR715789	(Zhang et al. 2023)
*M. phyllosaprophyticum*	SAUCC 3583-6	OR702572	OR702581	OR715796	OR715790	(Zhang et al. 2023)
*M. phragmitis*	CBS 285.71*	KP859013	KP858949	KP859077	KP859122	(Hernández-Restrepo et al. 2016)
*M. phragmitis*	CBS 423.78	KP859012	KP858948	KP859076	KP859121	(Hernández-Restrepo et al. 2016)
*M. poae*	LC12114*	MH740898	-	MH740914	MH740906	(Liang et al. 2019)
*M. ratticaudae*	BRIP 68298*	MW481661	MW481666	-	MW626890	(Crous et al. 2021a, b)
*M. rhopalostylidis*	CBS 145125*	MK442592	MK442532	-	MK442667	(Crous et al. 2019)
*M. salmonicolor*	KCTC 56427	NR173378	MK836108	-	-	(Das et al. 2020)
*M. seminicola*	KAS1516	KP859025	KP858961	KP859088	KP859134	(Hernández-Restrepo et al. 2016)
*M. seminicola*	KAS3576	KP859038	KP858974	KP859101	KP859147	(Hernández-Restrepo et al. 2016)
*M. shilinense*	CGMCC 3.23531*	OP103972	OP104022	OP242834	-	(Gao et al. 2022)
*M. sichuanense*	KUNCC23-13008*	OQ616510	OQ616434	-	OQ623473	(Dissanayake et al. 2023)
*M. sinense*	SAUCC 211097*	OM956289	OM959225	OM981144	OM981151	(Liu et al. 2022)
*M. sinense*	SAUCC 211098	OM956290	OM959226	OM981145	OM981152	(Liu et al. 2022)
*M. sorghi*	CBS 691.96	KP859000	KP858936	KP859063	KP859109	(Hernández-Restrepo et al. 2016)
*Microdochium* sp.	YNE01043	PP111924	PP111931	PP112584	PP112591	(Yan and Zhang 2024)
*Microdochium* sp.	YNE01771	PP111927	PP111934	PP112587	PP112592	(Yan and Zhang 2024)
*Microdochium* sp.	ZJ40	KJ572190	-	-	-	From NCBI
*M. streetiae*	BRIP 74742a*	OR947072	OR947079	-	-	(Tan and Shivas 2023b)
*M. streetiae*	BRIP 74752a	OR947071	OR947078	-	-	(Tan and Shivas 2023b)
*M. tainanense*	CBS 269.76*	KP859009	KP858945	KP859072	KP859118	(Hernández-Restrepo et al. 2016)
*M. tainanense*	CBS 270.76	KP858995	KP858931	KP859058	KP859104	(Hernández-Restrepo et al. 2016)
*M. trichocladiopsis*	CBS 623.77*	KP858998	KP858934	KP859061	KP859107	(Vu et al. 2019)
*M. triticicola*	RR 241	AJ748691	-	-	-	(Kwasna and Bateman 2007)
*M. yunnanense*	SAUCC 1011*	MT199881	MT199875	MT435650	MT510547	(Huang et al. 2020)
*M. yunnanense*	SAUCC 1015	MT199883	MT199877	MT435652	MT510549	(Huang et al. 2020)
*Peglionia verticiclada*	CBS 127654*	ON400763	ON400815	-	ON399352	(Hernández-Restrepo et al. 2022)
*Selenodriella brasiliana*	CBS 140227*	ON400769	ON400821	-	ON399356	(Hernández-Restrepo et al. 2022)
*Selenodriella cubensis*	CBS 683.96*	KP859053	KP858990	-	-	(Hernández-Restrepo et al. 2016)
*Selenodriella fertilis*	CBS 772.83	KP859055	KP858992	-	-	(Hernández-Restrepo et al. 2016)
*Selenodriella fertilis*	CPC 16273	ON400771	ON400823	-	ON399358	(Crous et al. 2019)
*Muscodor fengyangensis*	CGMCC 2862*	HM034856	HM034859	HM034843	HM034849	(Zhang et al. 2010)
*Muscodor thailandicus*	MFLUCC 17-2669*	MK762707	MK762714	MK776960	MK791283	(Samarakoon et al. 2020)

Notes: Species established in this study are shown in bold. Those marked “*” in the table are represented as ex-type or ex-epitype strains. “-’’: Not available.

### ﻿Phylogenetic analyses

In this study, 32 newly generated sequences were submitted to the NCBI GenBank database, and available reference sequences of *Microdochium* species were retrieved from GenBank for phylogenetic analysis. Sequences newly generated in this study were aligned alongside relevant sequences obtained from GenBank via the online MAFFT 7 tool implemented with the Auto strategy (Katoh et al. 2019; http://mafft.cbrc.jp/alignment/server/), followed by manual refinement using BioEdit (Hall 2006). To ascertain the species affiliation of the isolates, initial phylogenetic analyses were carried out for each locus individually, which was subsequently followed by a concatenated phylogenetic analysis incorporating all four loci (ITS, LSU, *rpb2*, and *tub2*). Phylogenetic analyses were conducted on both the individual and concatenated alignments of ITS, LSU, *rpb2*, and *tub2* sequences using maximum likelihood (ML) and Bayesian inference (BI) algorithms. Prior to BI analysis, the optimal evolutionary model for each partition was determined using MrModelTest v. 2.3 based on the Akaike Information Criterion (AIC), and the selected models were integrated into the analytical framework. ML and BI analyses were performed via either the CIPRES Science Gateway portal (https://www.phylo.org/, accessed on 30 June 2025) or offline software: ML was run using RAxML-HPC2 on XSEDE v. 8.2.12, and BI was conducted using MrBayes v. 3.2.7a on Linux with 64 threads. ML analysis used default parameters and was run with the GTR+G+I substitution model for 1,000 rapid bootstrap replicates, while BI utilized a rapid bootstrapping algorithm combined with an automatic stop feature. Finally, the resulting phylogenetic trees were visualized using FigTree v. 1.4.4 (http://tree.bio.ed.ac.uk/software/figtree, accessed on 30 June 2025) or ITOL: Interactive Tree of Life (https://itol.embl.de/, accessed on 30 June 2025), and final tree layouts were refined using Adobe Illustrator CC 2019.

## ﻿Results

### ﻿Phylogenetic analyses

**Figure 1.**
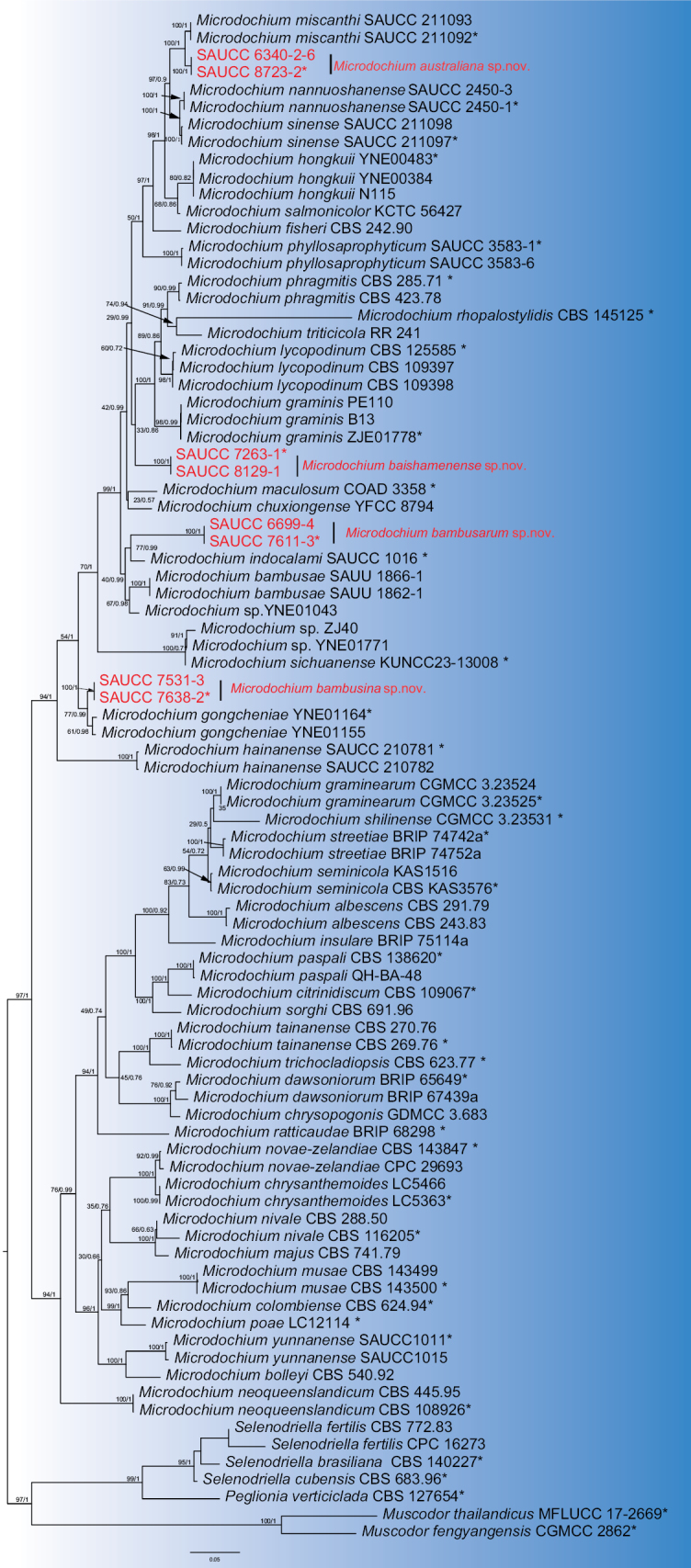
Maximum likelihood inference tree based on a combined dataset of analyzed ITS, LSU, *rpb2*, and *tub2* sequences. The Bayesian inference posterior probability (left, BIPP ≥ 0.90) and the maximum likelihood bootstrap value (right, MLBV ≥ 75%) are shown as BIPP/MLBV above the nodes. Those marked “*” in the tree are represented as ex-type or ex-epitype strains. Strains isolated in this study are indicated in red. The scale bar at the bottom indicates 0.07 substitutions per site. To enhance the visual appeal of the evolutionary tree layout, certain branches are shortened by two diagonal lines (“//”) with the number of times.

A phylogenetic analysis of *Microdochium* strains included a total of 80 sequences, with *Muscodor fengyangensis* (CGMCC 2862) and *Muscodor thailandicus* (MFLUCC 17-2669) as outgroups and sequences of *Selenodriella* and *Peglionia* as sister groups. The final concatenated alignment comprised 3,070 characters, viz. 1–681 (ITS), 682–1,515 (LSU), 1,516–2,369 (*rpb2*), and 2,370–3,274 (*tub2*). The final maximum likelihood (ML) log likelihood was 26959.183878. The matrix contained 1,300 distinct alignment patterns, with 27.86% of characters or gaps remaining undetermined. Estimated base frequencies were A = 0.219963, C = 0.292052, G = 0.257504, and T = 0.230481; substitution rates were AC = 1.126641, AG = 4.443240, AT = 1.324086, CG = 0.757689, CT = 6.915959, and GT = 1.000000. The alignment contained 1,304 unique site patterns (ITS: 315, LSU: 148, *rpb2*: 430, *tub2*: 411). The topology of the ML tree was congruent with that of the BI tree; thus, only the ML tree is shown (Fig. 1). The present study identified four novel species, viz. *Microdochium australiana* sp. nov., *M. baishamenense* sp. nov., *M. bambusina* sp. nov., and *M. bambusarum* sp. nov.

### ﻿Taxonomy

#### ﻿ Microdochium australiana

Taxon classificationFungiAmphisphaerialesAmphisphaeriaceae

Y.X. Shang, Z. Li & X.G. Zhang sp. nov.

FF4ABEE2-5371-5BAE-83B2-AD0EF7E34723

857092

Fig. 2

##### Etymology.

Referring to the species name of the host plant, *Phragmites australis*.

##### Holotype.

HSAUP 6340-2-6.

##### Description.

On leaves of *Phragmites australis*, Mycelia superficial and immersed, 2.2–2.9 µm wide, branched, membranous, hyaline. ***Conidiophores*** straight or slightly curved, aseptate, aggregated in the aerial mycelium, often reduced to conidiogenous cells borne directly from the hyphae. ***Conidiogenous cells*** terminal or intercalary, transparent, smooth, cylindric-clavate, 6.2–7.3 × 1.9–3.5 µm. ***Conidia*** solitary, cylindrical, ampulliform, 8.2–10.1 × 3.0–4.9 µm, multi-guttulate, 0–2-septate, apex rounded, base usually flattened. Sporodochia and chlamydospores not observed.

##### Culture characteristics.

Cultures incubated on PDA at 25 °C in darkness, reaching 48–50 mm diam, had a growth rate of 6.9–7.1 mm/day after 7 days. The center has obvious milky white aerial mycelium bulge, and the edge grayish, mycelium fluffy, back fawn.

##### Material examined.

China • Hainan Province, Jianfengling National Forest Park, on leaves of *Phragmites australis*, 26 June 2024, Y.X. Shang, (HSAUP 6340-2-6), ex-holotype culture SAUCC 6340-2-6 = CGMCC 3.28622; Ibid., (HSAUP 8723-2, paratype), living culture SAUCC 8723-2.

**Figure 2.**
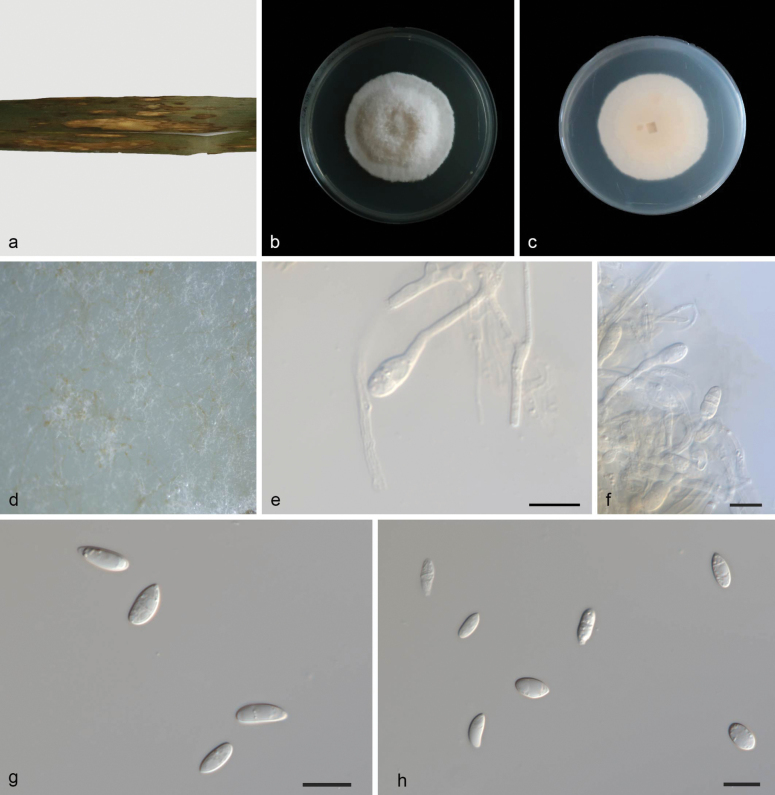
*Microdochium australiana* (CGMCC 3.28622, ex-holotype culture). a. A leaf of *Phragmites australis*; b, c. Colonies on PDA from above and below after 7 days; d. Colony overview; e, f. Conidiogenous cells and conidia; g, h. Conidia. Scale bars: 10 μm (e–h).

##### Notes.

*Microdochium australiana* is closely related to *M. miscanthi* (SAUCC211092 and SAUCC211093) based on DNA sequence data in BLAST searches and phylogenetic analysis (Fig. 1). However, *M. australiana* differs from *M. miscanthi* by 28 nucleotides (1/541 in ITS, 1/866 in LSU, 13/730 in *tub2*, and 13/665 in *rpb2*). In morphology, they are distinguished by different hosts (*Phragmites australis* vs. *Miscanthus sinensis*), and *M. australiana* colonies on PDA exhibit a prominent, milky white aerial mycelium bulge, with a central pale orange region and a grayish edge. In contrast, *M. miscanthi* colonies are overall white, featuring a central dark-green plaque covered by white mycelia. Mycelial width (2.2–2.9 µm) in *M. australiana* vs. (1.5–2.3 µm) in *M. miscanthi*. Conidia in *M. australiana* differ from those in *M. miscanthi* (cylindrical, ampulliform vs. transparent, spindle-to-rod-shaped) (Liu et al. 2022). Therefore, we establish this fungus as *M. australiana* sp. nov.

#### ﻿ Microdochium baishamenense

Taxon classificationFungiAmphisphaerialesAmphisphaeriaceae

Y.X. Shang, Z. Li & X.G. Zhang sp. nov.

8497FBBE-0666-5FC3-8DA5-43409241E5E1

857094

Fig. 3

##### Etymology.

The epithet “*baishamenense*” is named after Baishamen Park, where the fungus was collected.

##### Holotype.

HSAUP 8129-1.

##### Type.

China, Hainan Province, Baishamen Park, on leaves of Bambusaceae sp., 24 June 2024, Y.X. Shang, (HSAUP 8129-1), ex-holotype culture SAUCC 8129-1 = CGMCC 3.28625.

##### Description.

On leaves of Bambusaceae sp. Mycelium superficial and immersed; hyphae hyaline, branched, septate. ***Conidiophores*** slightly differentiated, bifurcate, hyaline, smooth. ***Conidiogenous cells*** terminal, sympodial, denticulate, cylindrical, 19–60 × 1.5–2 μm, hyaline, smooth. ***Conidia*** soli tary, dry, fusiform, obovoid, subpyriform, to clavate, 7–12 × 3–4 μm, 0–1-septate, hyaline, tapering to a subtruncate hilum; hilum unpigmented. Chlamydospores not observed.

##### Culture characteristics.

Cultures incubated on PDA at 25 °C in darkness, reaching 46–51 mm diam, had a growth rate of 6.5–7.2 mm/day after 7 days. The center has milky white, mycelia lush and the edge mycelium was sparse, pale brown, back light yellow.

##### Material examined.

China • Hainan Province, Baishamen Park, on leaves of Bambusaceae sp., 24 June 2024, Y.X. Shang, (HSAUP 8129-1), ex-holotype culture SAUCC 8129-1 = CGMCC 3.28625; Ibid., (HSAUP 7263-1, paratype), living culture SAUCC 7263-1.

**Figure 3.**
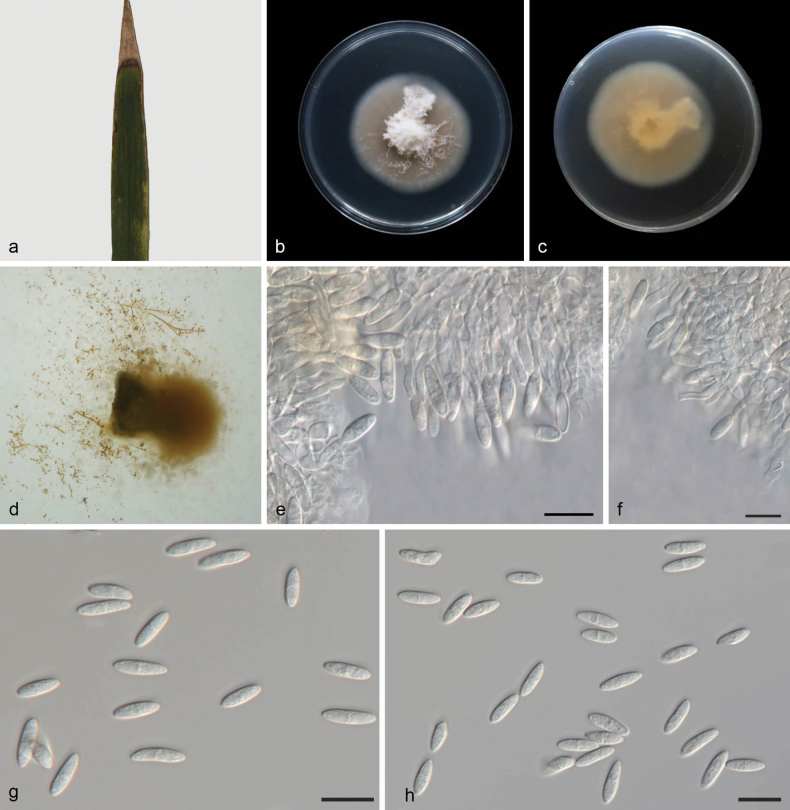
*Microdochium baishamenense* (CGMCC 3.28625, ex-holotype culture). a. A leaf of *Phragmites australis*; b, c. Colonies on PDA from above and below after 7 days; d. Colony overview; e, f. Conidiogenous cells and conidia; g, h. Conidia. Scale bars: 10 μm (e–h).

##### Notes.

Phylogenetic analysis showed that *Microdochium baishamenense* formed an independent clade, where it shows a relationship with *M. fisheri* (CBS 242.90). *M. baishamenense* differs from *M. fisheri* (CBS 242.90) by its production of shorter conidiogenous cells (4.5–7.3 × 1.5–2.4 µm vs. 19–60 × 1.5–2 μm) and distinct morphological features. Specifically, the center of *M. baishamenense* colonies is milky white, with lush mycelia; the mycelia at the edge are sparse and pale brown, and the colony reverse is light yellow. In contrast, *M. fisheri* (CBS 242.90) exhibits a salmon-colored center, a peach-colored periphery, and an entire margin. *M. baishamenense* produced cylindrical, clavate to obovoid conidia, whereas *M. fisheri* (CBS 242.90) has conidia that are tarry, dry, fusiform, obovoid, or subpyriform, and there are 155 distinct nucleotide positions separating the species (14/527 in ITS, 13/830 in LSU, 34/687 in *tub2*, 94/839 in *rpb2*) (Hernández-Restrepo et al. 2016).

#### ﻿ Microdochium bambusina

Taxon classificationFungiAmphisphaerialesAmphisphaeriaceae

Y.X. Shang, Z. Li & X.G. Zhang sp. nov.

E4EF98D5-23DA-5083-933A-91E4D0644404

857091

Fig. 4

##### Etymology.

Referring to the species name of the host plant, Bambusaceae sp.

##### Holotype.

HSAUP 7531-3.

##### Description.

Parasitic on leaves of Bambusaceae sp. Mycelia superficial and immersed, 2.2–3.5 µm wide, transparent, smooth, branched, diaphragmatic. ***Conidiophores*** indistinct, often reduced to conidiogenous cells. ***Conidiogenous cells*** straight or bent, smooth, cylindrical, 2.3–3.8 × 1.2–2.0 µm. ***Conidia*** solitary, hyaline, ellipsoid, spindle‐to‐rod‐shaped, 4.8–7.2 × 2.1–3.4 µm, multi-guttulate. Sporodochia and chlamydospores not observed.

##### Culture characteristics.

Cultures incubated on PDA at 25 °C in darkness, reaching 33–36 mm diam, had a growth rate of 4.7–5.1 mm/day after 7 days. They are circular, white and dense in the center, yellowish and flocculent at the edge.

##### Material examined.

China • Hainan Province, Diaoluo Mountain, on leaves of Bambusaceae sp., 24 June 2024, Y.X. Shang, (HSAUP 7531-3), ex-holotype culture SAUCC 7531-3 = CGMCC 3.28623; Ibid., (HSAUP 7638-2, paratype), living culture SAUCC 7638-2.

##### Notes.

*Microdochium bambusina* is closely related to *M. gongcheniae* (YNE01164) based on DNA sequence data in BLAST searches and phylogenetic analysis. *M. bambusina* is distinguished from *M. gongcheniae* (YNE01164) by 5/534, 1/926, and 8/540 characters in ITS, LSU, and *tub2* sequences, respectively. In morphology, they are distinguished by different hosts (Bambusoideae sp. vs. *Paspalum* sp.). The two species exhibit significant morphological divergence. *M. bambusina* produces conidia measuring 4.8–7.2 × 2.1–3.4 μm, which are significantly larger than those of *M. gongcheniae* (3.3–6.4 × 0.8–2.3 μm), and *M. bambusina* colonies on PDA are gray white with regular margins. *M. gongcheniae* colonies on PDA are orange with flocculent mycelia, and subtle differences are observed in the shapes of conidia: *M. bambusina* conidia are ellipsoid, spindle-to-rod-shaped, whereas *M. gongcheniae* exhibits reniform to fusiform conidia (Yan and Zhang 2024). Therefore, based on morphology and phylogeny, we establish this fungus as *Microdochium bambusina* sp. nov.

**Figure 4.**
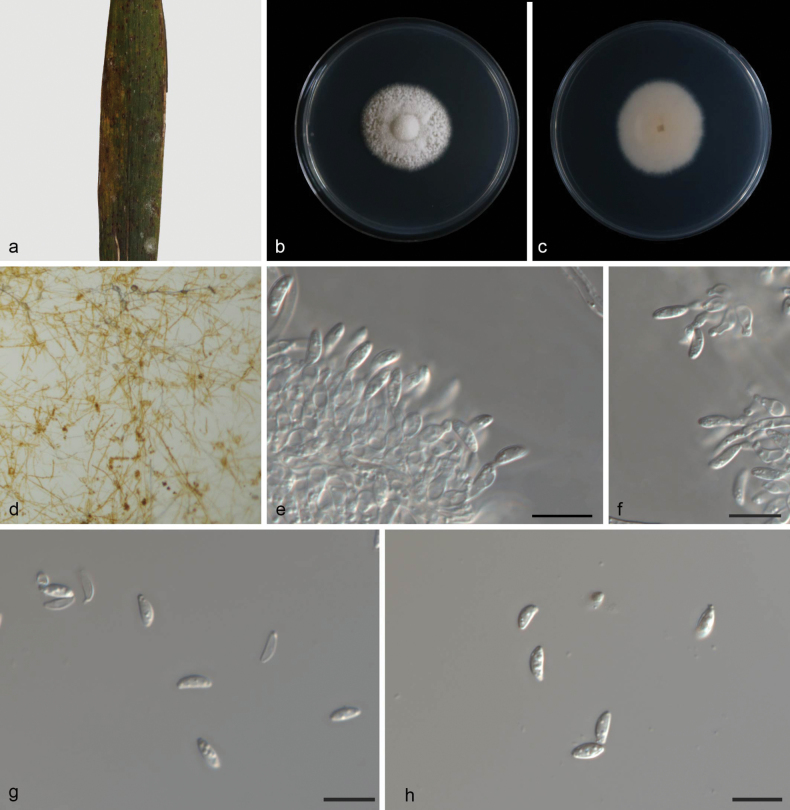
*Microdochium bambusina* (CGMCC 3.28623, ex-holotype culture). a. A leaf of Bambusaceae sp.; b, c. Surface and reverse sides of colony after 7 days on PDA; d. Colony overview with conidiomata; e, f. Conidiogenous cells with conidia; g, h. Conidia. Scale bars: 10 μm (e–h).

#### ﻿ Microdochium bambusarum

Taxon classificationFungiAmphisphaerialesAmphisphaeriaceae

Y.X. Shang, Z. Li & X.G. Zhang sp. nov.

49A7D93A-1DF1-5D07-8873-4B3C5BFAFEE2

857093

Fig. 5

##### Etymology.

Referring to the species name of the host plant, Bambusaceae sp.

##### Holotype.

HSAUP 7611-3.

##### Description.

Parasitic on leaves of Bambusaceae sp. Mycelium superficial and immersed; hyphae hyaline, septate, smooth, branched, 2.4–3.7 μm wide. ***Conidiogenous*** indistinct, often reduced to conidiogenous cells. ***Conidiogenous cells*** 6.3–7.0 × 2.0–2.8 µm, transparent, smooth, prismatic, fusoid. ***Septiceless conidia*** 10–11 × 4.2–4.5 μm, ellipsoid, hyaline, aseptate. ***Septate conidia*** 11.6–14.8 × 3.5–4.5 μm, solitary, lunate, ellipsoid, hyaline, straight or curved, obtuse, 2–3-septate.

##### Culture characteristics.

Cultures incubated on PDA at 25 °C in darkness, reaching 54–60 mm diam, had a growth rate of 7.7–8.6 mm/day after 7 days. The center has obvious milky white aerial mycelium bulge, and the edge mycelium was smooth, back light yellow.

##### Material examined.

China • Hainan Province, Diaoluo Mountain, on leaves of Bambusaceae sp., 26 June 2024, Y.X. Shang, (HSAUP 7611-3), ex-holotype culture SAUCC 7611-3 = CGMCC 3.28624; Ibid., (HSAUP 6699-4, paratype), living culture SAUCC 6699-4.

##### Notes.

*Microdochium bambusarum* is closely related to *M. indocalami* (SAUCC 1016) based on DNA sequence data in BLAST searches and phylogenetic analysis. However, *M. bambusarum* differs from *M. indocalami* (SAUCC 1016) by 39 nucleotides (11/528 in ITS, 3/886 in LSU, 19/840 in *rpb2*, and 6/727 in *tub2*). Morphologically, they are distinguished by different hosts (Bambusoideae sp. vs. *Indocalamus longiauritus*), and *M. bambusarum* colonies on PDA are gray white with regular margins. In *M. bambusarum*, conidiogenous cells are prismatic and fusoid, whereas in *M. indocalami* they are cylindrical, straight, or bent (Huang et al. 2020). Therefore, we define these two isolates as a new species.

**Figure 5.**
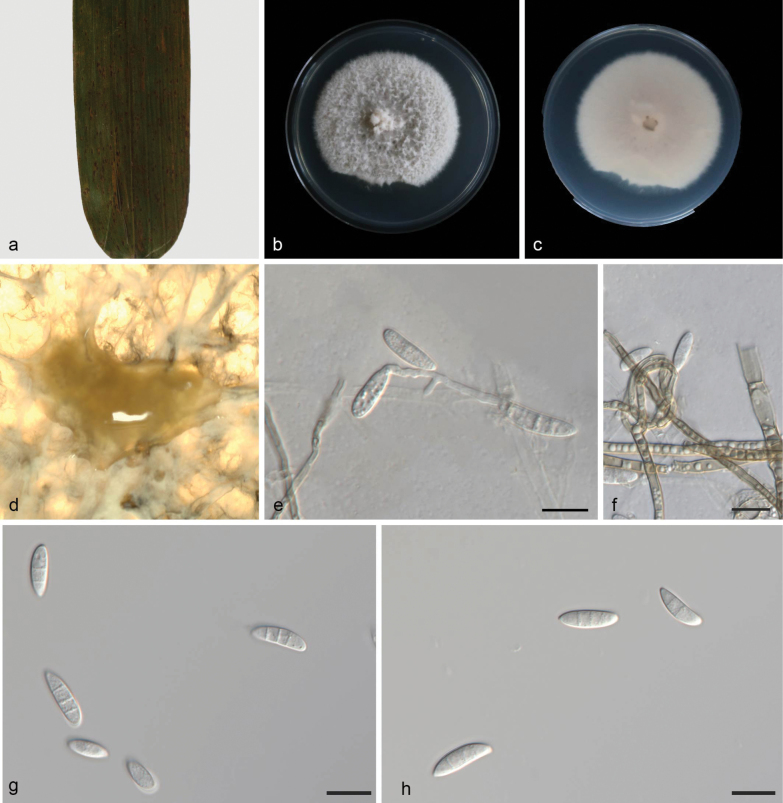
*Microdochium bambusarum* (CGMCC 3.28624, ex-holotype culture). a. A leaf of Bambusaceae sp.; b, c. Colonies on PDA from above and below after 7 days; d. Colony overview; e, f. Conidiogenous cells and conidia; g, h. Conidia. Scale bars: 10 μm (e–h).

## ﻿Discussion

Recent studies have described four new species (*Microdochium australiana*, *M. baishamenense*, *M. bambusina*, and *M. bambusarum*) isolated from two host plants: Bambusaceae sp. and *Phragmites australis*. To date, the Global Biodiversity Information Facility (GBIF; https://www.gbif.org/; data accessed 30 June 2024) has recorded 1,879 georeferenced occurrences of *Microdochium* species worldwide, concentrated primarily in temperate, subtropical, and tropical regions, particularly in the Americas, Asia, Europe, and Oceania. The United States hosts the highest concentration, followed by Australia, China, New Zealand, and Poland. In China, *Microdochium* has been reported in Anhui, Gansu, Henan, Jiangsu, Shandong, Shanxi, Sichuan, Yunnan, and Zhejiang, among other regions. The genus has a broad global distribution but shows a higher frequency of occurrences in warm, humid regions, and its members mostly parasitize grasses. The discovery of these four new species further confirms the strong graminicolous (grass-parasitizing) habit of the genus. These species were collected in Hainan Province, characterized by a tropical rainforest climate with an annual average temperature of 22–27 °C and annual precipitation of 1,000–2,600 mm. This warm, humid environment is highly suitable for *Microdochium* growth, aligning with its observed distribution pattern.

To achieve robust species delimitation amid morphologically conserved traits, a combined phylogenetic approach was employed using four molecular markers: ITS, LSU, *rpb2*, and *tub2*. While LSU offers sufficient variation for generic-level assignment, the concatenation of multiple loci significantly enhances resolution for species-level identification, reducing taxonomic ambiguity and facilitating the recognition of phylogenetically distinct taxa (Crous et al. 2021b). The adoption of the guidelines proposed by Hernández-Restrepo et al. (2016) further ensures that species validation meets contemporary taxonomic standards, integrating morphological nuance with molecular phylogenetic support.

This study aims to elucidate the distribution and host diversity of *Microdochium*, providing taxonomic foundations for clarifying its evolutionary relationships.

## Supplementary Material

XML Treatment for Microdochium australiana

XML Treatment for Microdochium baishamenense

XML Treatment for Microdochium bambusina

XML Treatment for Microdochium bambusarum

## ﻿Additional information

### Use of AI

No use of AI was reported.

### Author ORCIDs

Yuxin Shang https://orcid.org/0009-0000-7211-5139

Qiyun Liu https://orcid.org/0009-0009-9545-7962

Zhaoxue Zhang https://orcid.org/0000-0002-4824-9716

Mengyuan Zhang https://orcid.org/0009-0002-3068-7491

Duhua Li https://orcid.org/0009-0006-5200-2034

Xiuguo Zhang https://orcid.org/0000-0001-9733-8494

Jiwen Xia https://orcid.org/0000-0002-7436-7249

Zhuang Li https://orcid.org/0000-0003-0583-0399

### Data availability

All of the data that support the findings of this study are available in the main text or Supplementary Information.

## Supplementary materials

Supplementary material 1 Phylogeny of the *Microdochium* species complex Data type docx Explanation note Phylogeny of the *Microdochium* species complex (FIESC) inferred based on the ITS (**a**), LSU (**b**), *rpb2* (**c**), and *tub2* (**d**) loci, respectively. https://binary.pensoft.net/file/1492803This dataset is made available under the Open Database License (http://opendatacommons.org/licenses/odbl/1.0/). The Open Database License (ODbL) is a license agreement intended to allow users to freely share, modify, and use this Dataset while maintaining this same freedom for others, provided that the original source and author(s) are credited.Yuxin Shang, Qiyun Liu, Zhaoxue Zhang, Mengyuan Zhang, Zixu Dong, Duhua Li, Yaling Wang, Congcong Ai, Xiuguo Zhang, Jiwen Xia, Zhuang Li
