# Supplementary material for: ﻿Four new species of Microdochium (Microdochiaceae, Xylariales) from Hainan, China
Source: MycoKeys. 2025 Dec 15;126:151–70. doi: 10.3897/mycokeys.126.170451 (PMC12723394; doi:10.3897/mycokeys.126.170451)
Supplement: Supplementary material 1 — Phylogeny of the Microdochium species complex [file mycokeys-126-151-s001.docx]

(A)


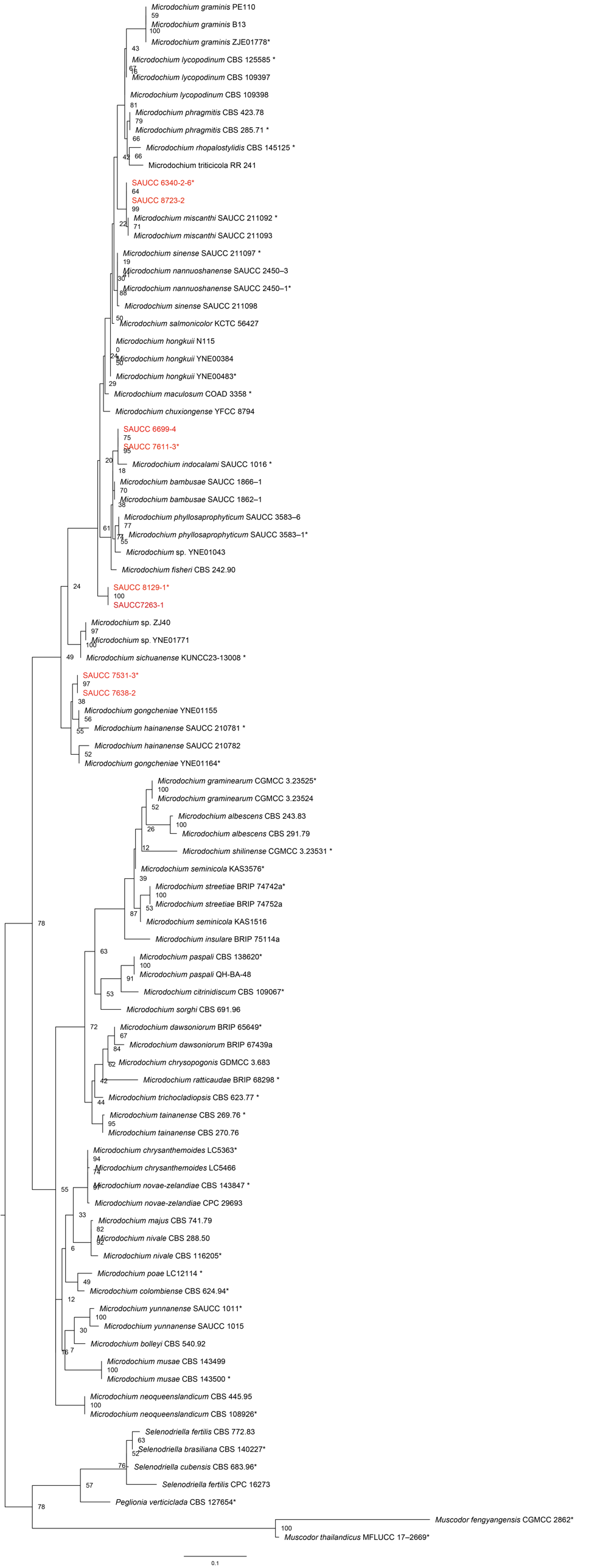


Fig. S1A: Phylogeny of the Microdochium species inferred based on the ITS (a)，with *Muscodor* as the outgroup taxa. The RAxML Bootstrap support values (ML-BS > 70 %) were displayed at the nodes. Those marked “*” in the tree are represented as ex-type or ex-epitype strains Strains isolated in this study were indicated in red.


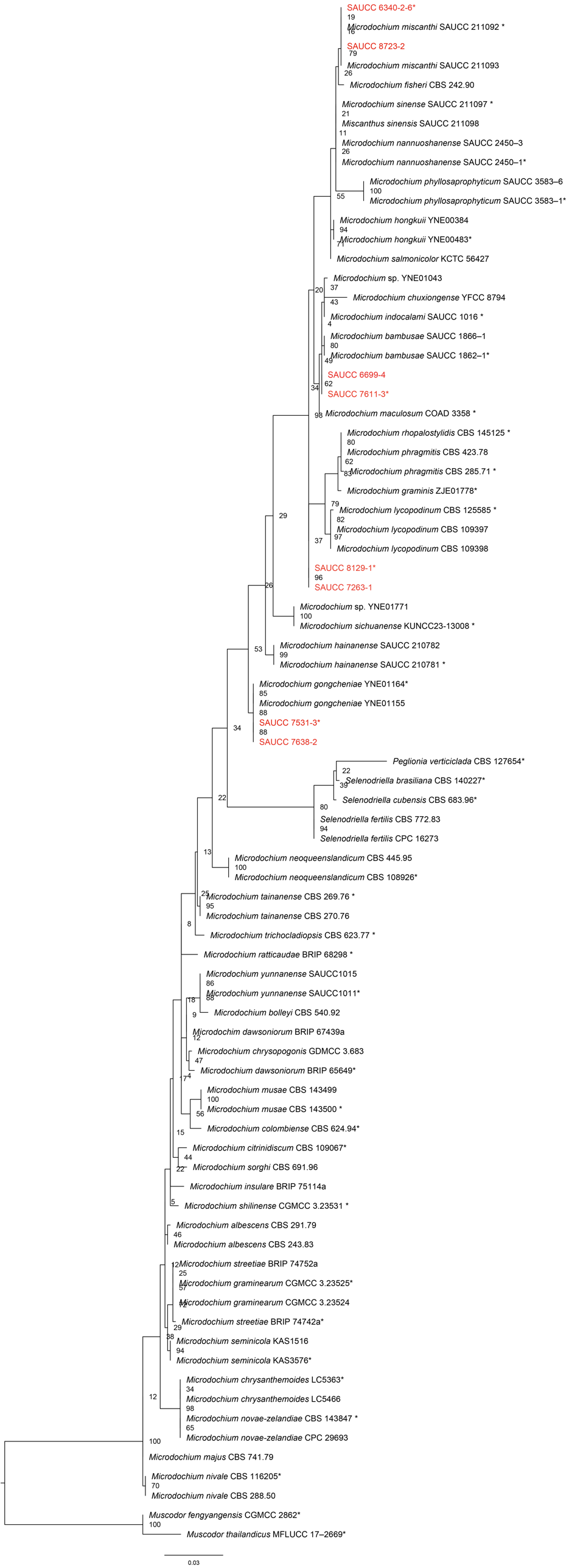


(B)

Fig. S1B: Phylogeny of the Microdochium species inferred based on the LSU (B)，with *Muscodor*, *Selenodriella and Peglionia* as the outgroup taxa. The RAxML Bootstrap support values (ML-BS > 70 %) were displayed at the nodes. Those marked “*” in the tree are represented as ex-type or ex-epitype strains Strains isolated in this study were indicated in red.


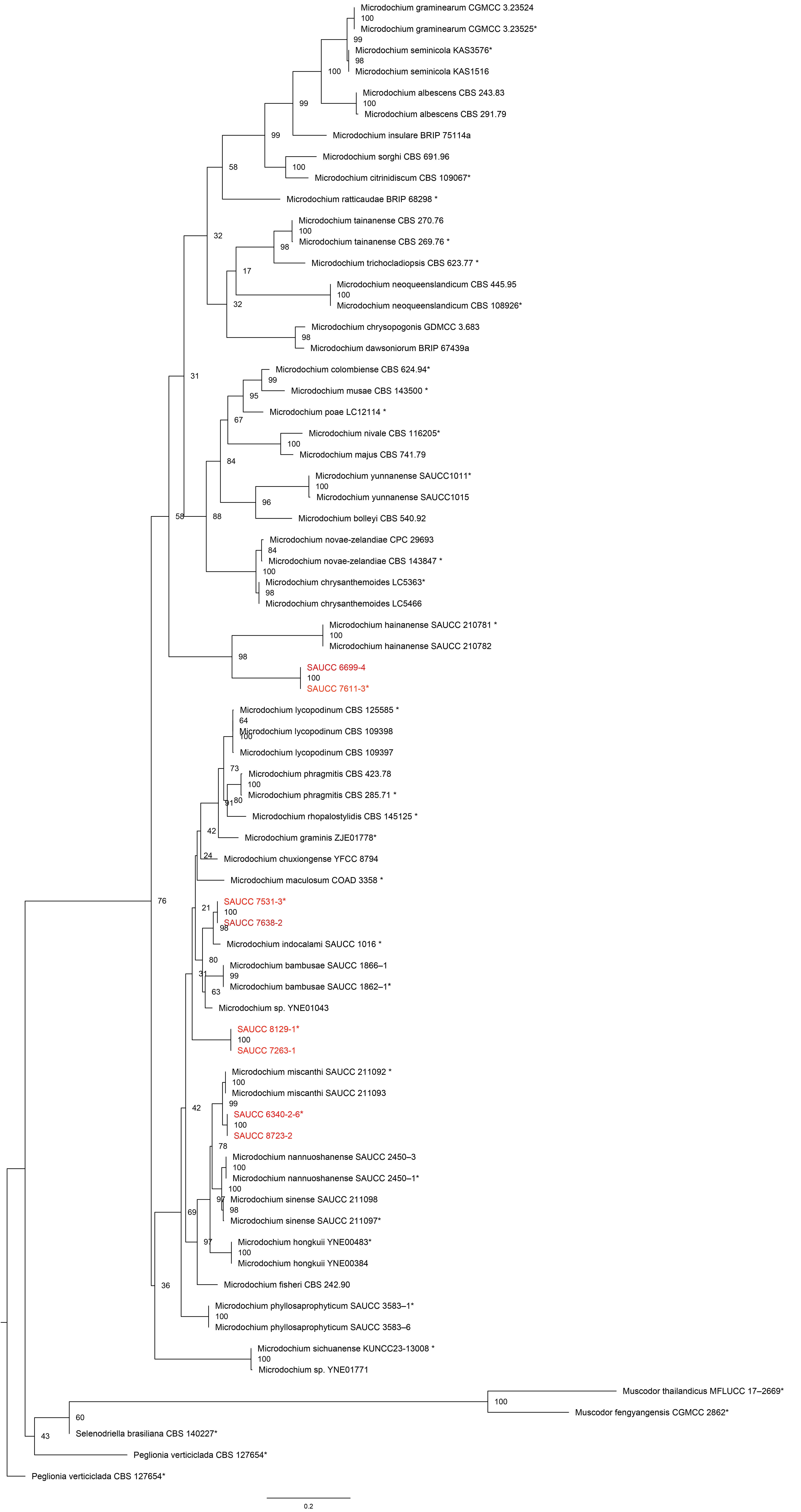


(C)

Fig. S3C: Phylogeny of the Microdochium species inferred based on the *rpb2* (c)，with *Muscodor*,*Selenodriella and Peglionia* as the outgroup taxa. The RAxML Bootstrap support values (ML-BS > 70 %) were displayed at the nodes. Those marked “*” in the tree are represented as ex-type or ex-epitype strains Strains isolated in this study were indicated in red.

(D)


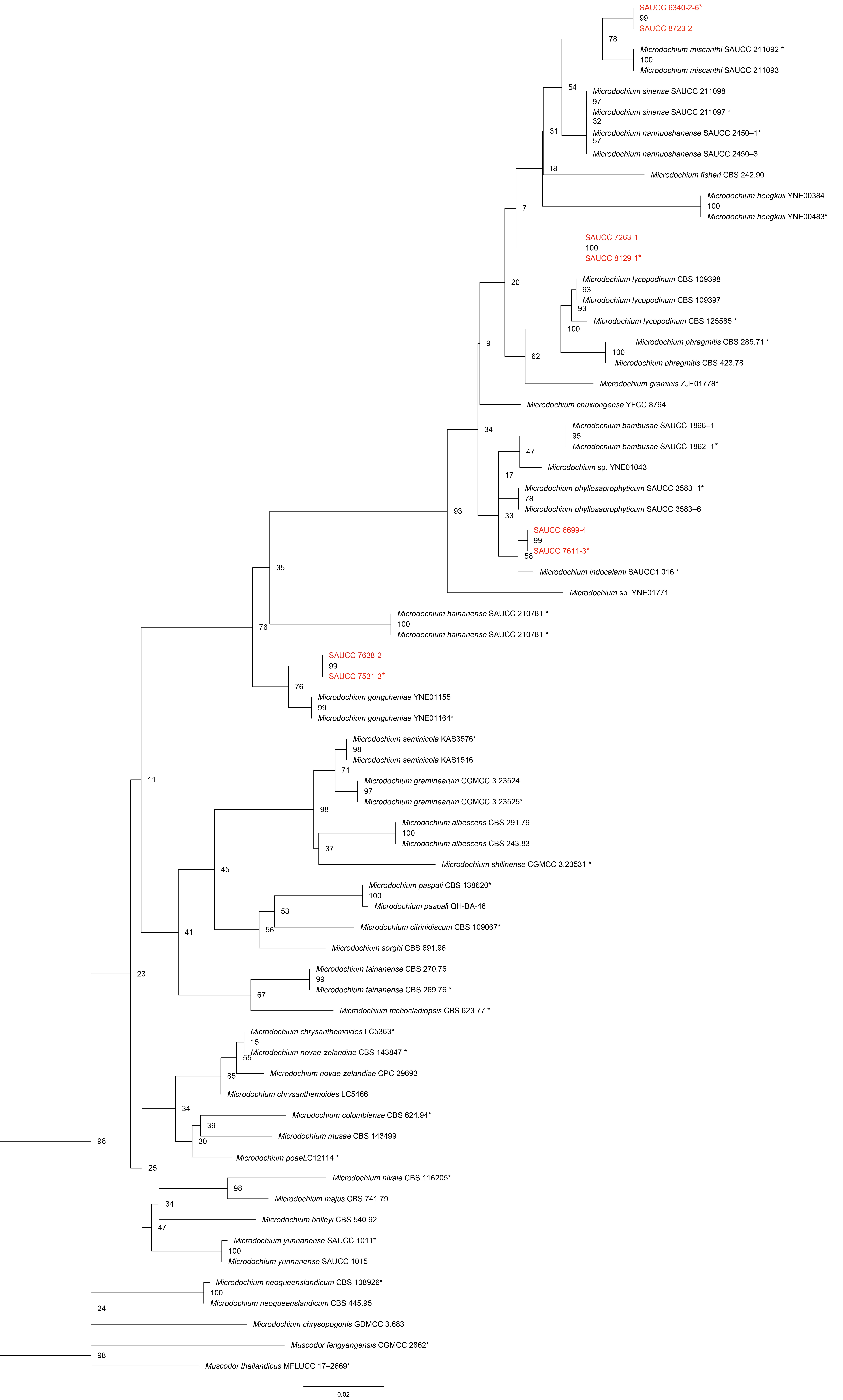


Fig. S4D: Phylogeny of the Microdochium species inferred based on the *tub2* (d)，with *Muscodor* as the outgroup taxa. The RAxML Bootstrap support values (ML-BS > 70 %) were displayed at the nodes. Those marked “*” in the tree are represented as ex-type or ex-epitype strains Strains isolated in this study were indicated in red.
